# From species to communities: the signature of recreational use on a tropical river ecosystem

**DOI:** 10.1002/ece3.1800

**Published:** 2015-11-12

**Authors:** Amy E. Deacon, Hideyasu Shimadzu, Maria Dornelas, Indar W. Ramnarine, Anne E. Magurran

**Affiliations:** ^1^ Centre for Biological DiversityUniversity of St AndrewsSt AndrewsFifeUnited Kingdom; ^2^ Department of Life SciencesThe University of the West IndiesSt AugustineTrinidad and Tobago

**Keywords:** Allocation rules, anthropogenic disturbance, biodiversity, community capacity, diversity indices, ecosystem services, freshwater ecology, *Poecilia reticulata*

## Abstract

Disturbance can impact natural communities in multiple ways. However, there has been a tendency to focus on single indicators of change when examining the effects of disturbance. This is problematic as classical diversity measures, such as Shannon and Simpson indices, do not always detect the effects of disturbance. Here, we instead take a multilevel, hierarchical approach, looking for signatures of disturbance in the capacity and diversity of the community, and also in allocation and demography at the population level. Using recreational use as an example of disturbance, and the freshwater streams of Trinidad as a model ecosystem, we repeatedly sampled the fish communities and physical parameters of eight pairs of recreational and nonrecreational sites every 3 months over a 28‐month period. We also chose the Trinidadian guppy (*Poecilia reticulata*) as the subject of our population‐level analyses. Regression tree analysis, together with analysis of deviance, revealed that community capacity and community species richness were greater at sites with higher levels of recreational use. Interestingly, measures of community diversity that took into account the proportional abundance of each species were not significantly associated with recreational use. Neither did we find any direct association between recreational use and proportion of guppy biomass in the community. However, population‐level differences were detected in the guppy: Sex ratio was significantly more female‐biased at more disturbed sites. Our findings emphasize the importance of considering multiple levels when asking how disturbance impacts a community. We advocate the use of a multilevel approach when monitoring the effects of disturbance, and highlight gaps in our knowledge when it comes to interpreting these effects.

## Introduction

Understanding the consequences of both human and natural forms of disturbance is a key challenge in community ecology (Connell 1978; Wootton 1998; White and Jentsch 2001; Dornelas et al. 2011b). Although natural disturbance has always been a component of the environment in which communities of species have evolved, anthropogenic disturbance is now occurring with such prevalence that there are calls to name the current time period the “Anthropocene.”

It is common for studies to focus on just one or two measures when attempting to detect the effects of disturbance. A problem with this is that the classical diversity measures (i.e., species richness; Shannon and Simpson indices) do not always detect disturbance (Magurran 2004; Dornelas et al. 2011b). Effects of disturbance do not have a consistent signature in these metrics, even when they cause marked changes in community composition because of compensatory dynamics (Supp and Ernest 2014). Moreover, disturbance can affect biodiversity in different ways, depending on the processes of community dynamics involved. The structure of a community, in terms of both species richness and species relative abundance, is a consequence of environmental filtering, ecological processes such as competition and predation, and dispersal limitation.

Disturbance can alter the environment and affect all of these processes and has the potential to impact communities at different hierarchical levels, from the fundamental property of capacity to the emergent property of species richness (Connell 1978; Huston 1979; Wootton et al. 1996; Kondoh 2001; Dornelas et al. 2011b; Murry and Farrell 2014). As outlined in Dornelas' (2010) conceptual approach, disturbance can affect community capacity (the total biomass or organismal abundance that a particular ecosystem can support [Brown 1981]) or the community demographic rates (mortality, fecundity, and migration). The latter controls how this capacity is *allocated* between species and individuals, and is reflected in the diversity patterns of a community. In every community, some species are common while the majority are rare (McGill et al. 2007), but the distribution of abundance among species can vary markedly among assemblages. Furthermore, human disturbance can also directly affect the constituent species in a community (Resh et al. 1988; Schlosser 1991; Lake et al. 2000; Agostinho et al. 2008). Given the complex nature of the interactions involved, the consequences of disturbance can be dynamic and difficult to predict (Lake et al. 2000; Kondoh 2001; Dornelas 2010).

One important source of anthropogenic disturbance is recreational use (Liddle and Scorgie 1980; Boyle and Samson 1985; Edington and Edington 1986; Newsome and Moore 2012). Forms of disturbance associated with recreation include harvesting, habitat modification, pollution, and noise impacts on wildlife (Knight and Gutzwiller 1995). These can have far‐reaching implications in terms of the productivity, physical parameters, and species composition of a site—primarily by affecting nutrient levels, water quality, and the behavior of inhabitants (Burt and Rice 2009; Rehnus et al. 2014) which, in turn, affect ecosystem services (Costanza et al. 1997). However, recreational use is an important ecosystem service in itself (Daniel et al. 2012) and can promote understanding and appreciation of the natural environment in those that utilize it (Mace et al. 2012) which ultimately may increase the likelihood that it will be conserved. Here, we quantify effects of recreational use on freshwater biodiversity and ask how human activity mediates a suite of informative community variables.

Freshwater habitats support 6% of all described species, an extraordinarily high value given that they account for less than 1% of the Earth's surface (Hawksworth and Kalin‐Arroyo 1995). Freshwater systems also provide essential ecosystem services including water purification, food production, and water supply for irrigation (Costanza et al. 1997), yet they are particularly at risk from anthropogenic change (Balmford et al. 2002; Dudgeon et al. 2006; Abell et al. 2008; WWF 2014).

Anthropogenic threats to freshwater ecosystems fall into five main categories: over‐exploitation, water pollution, flow modification, destruction and degradation, and biological invasions (Dudgeon et al. 2006). The use of rivers for recreation can contribute to all five of these categories through fishing, dumping of waste, construction of dams, improving accessibility, and increasing opportunities for spread of invasive species (Liddle and Scorgie 1980; Kaufman 1992; Primack 1992; Lake et al. 2000).

People tend to live near to rivers, increasing pressure on freshwater habitats (Sala et al. 2000; Revenga et al. 2005; Paul and Meyer 2001). In the tropics, this problem is intensified, with rivers and drainage basins even more densely populated than in temperate regions (Dudgeon et al. 2006). Indeed, rates of tropical freshwater biodiversity loss are estimated to be faster than for any other biome (Ricciardi and Joseph 1999; WWF 2014), making such habitats a priority when it comes to understanding the effects of disturbance on biodiversity and other ecosystem properties (Abell 2002; Dudgeon et al. 2006).

Located in a “hotspot” of freshwater biodiversity (Myers et al. 2000; Abell et al. 2008), Trinidad's Northern Range has provided scientists with a natural freshwater laboratory for ecological study for decades (Haskins and Haskins 1951; Reznick et al. 2008). Numerous parallel rivers descending its southern slopes offer an unrivaled opportunity for replicated experimental design (Magurran 2005). Unlike many tropical riverine habitats, the majority of these streams are easily accessible. This is convenient for scientists, but also means that some parts are intensively used by local inhabitants for recreation; known locally as a “river lime,” the act of spending a day picnicking and bathing in and around a river is popular and embedded in Trinidadian culture (Alkins‐Koo et al. 2004). Recreational sites tend to be localized, such that there are relatively untouched sections of stream nearby. The presence of these recreational–nonrecreational pairs of sites provides an opportunity to measure the impact of human recreational use on these irreplaceable aquatic communities.

**Figure 1:**
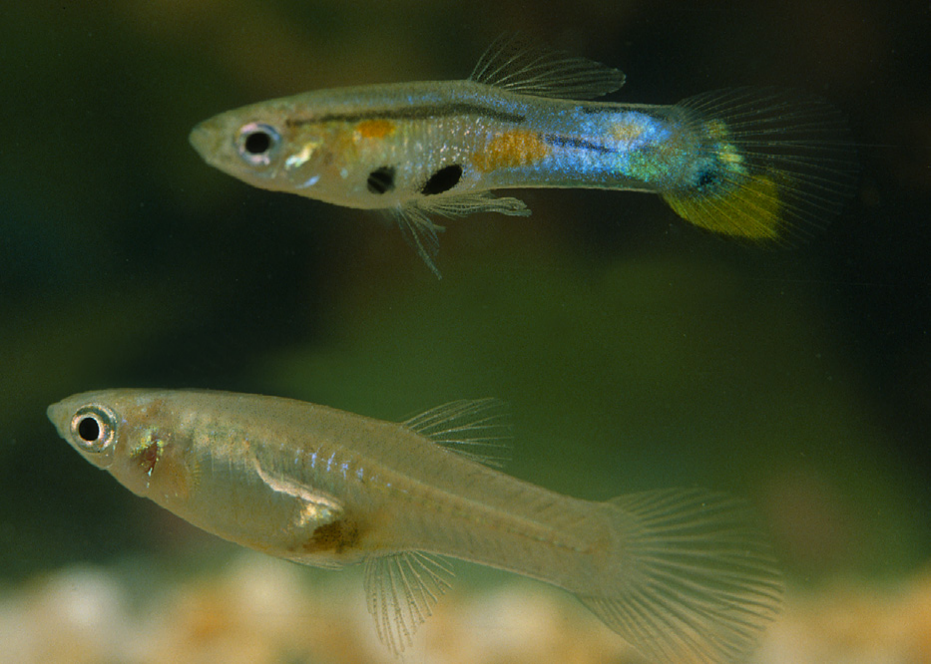
The Trinidadian guppy *(Poecilia reticulata)*, male (above), and female (below).

The guppy, *Poecilia reticulata*, is nearly ubiquitous in the waterways of Trinidad, and particularly abundant in the Northern Range (Fig. 1). Incredibly adaptable in terms of life‐history, behavior, and ecology, it is also a prolific breeder; a high rate of population turnover leads to variability in population structure, and the sex ratio of populations varies in response to predation risk and other factors (Rodd and Reznick 1997; Pettersson et al. 2004). Because of our existing knowledge of its ecology, its presence in multiple freshwater habitats, and its flexible life‐history, the guppy is an ideal species for exploring potential population‐level effects of disturbance in Trinidad's freshwater streams (Noss 1990; Magurran 2005).

**Figure 2:**
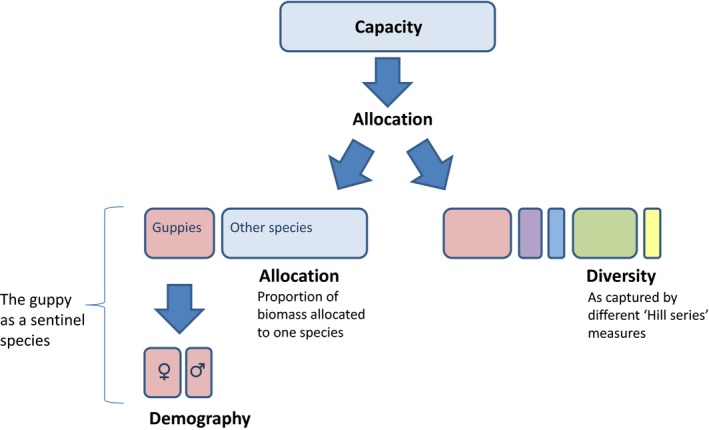
Hierarchical levels of community properties that can be explored in relation to disturbance.

Here, we examine the consequences of anthropogenic disturbance, in the form of recreation, on the multiple components of the freshwater communities in these Northern Range rivers. Using matched pair sites, we quantify the following: total available biomass (community capacity); the Hill series measures of diversity (Hill 1973): species richness, reciprocal of Simpson index, exponential Shannon index and Berger–Parker index (community diversity); the allocation of biomass to a single species, the guppy (community allocation); and intraspecific effects on the guppy (demography) (Fig. 2).

We predict that disturbance to freshwater habitats as a result of recreational use will be detectable at multiple levels of community properties. We predict that disturbance will be linked to an increase in community capacity, as the recreational activities associated with river use are likely to lead to rises in productivity through removal of canopy cover and the presence of discarded food (Lake et al. 2000; Hadwen and Bunn 2004). In addition, we compare a range of measures of community diversity in their detection of recreational disturbance. Finally, we focus on the demography of one component of the community, the guppy, and predict a shift in sex ratio in disturbed relative to control sites (Pettersson et al. 2004).

These predictions are tested by sampling the fish communities and physical characteristics at eight recreational–nonrecreational pairs of sites along the southern slopes of Trinidad's Northern Range ten times each during a 28‐month period. We use regression tree analysis to test our overarching hypothesis that the effects of disturbance are different for different community properties, and examine its consequences for capacity, four different measures of diversity, allocation, and guppy demography using generalized linear models (GLMs) and analysis of deviance.

## Materials and Methods

### Sampling methods

**Figure 3:**
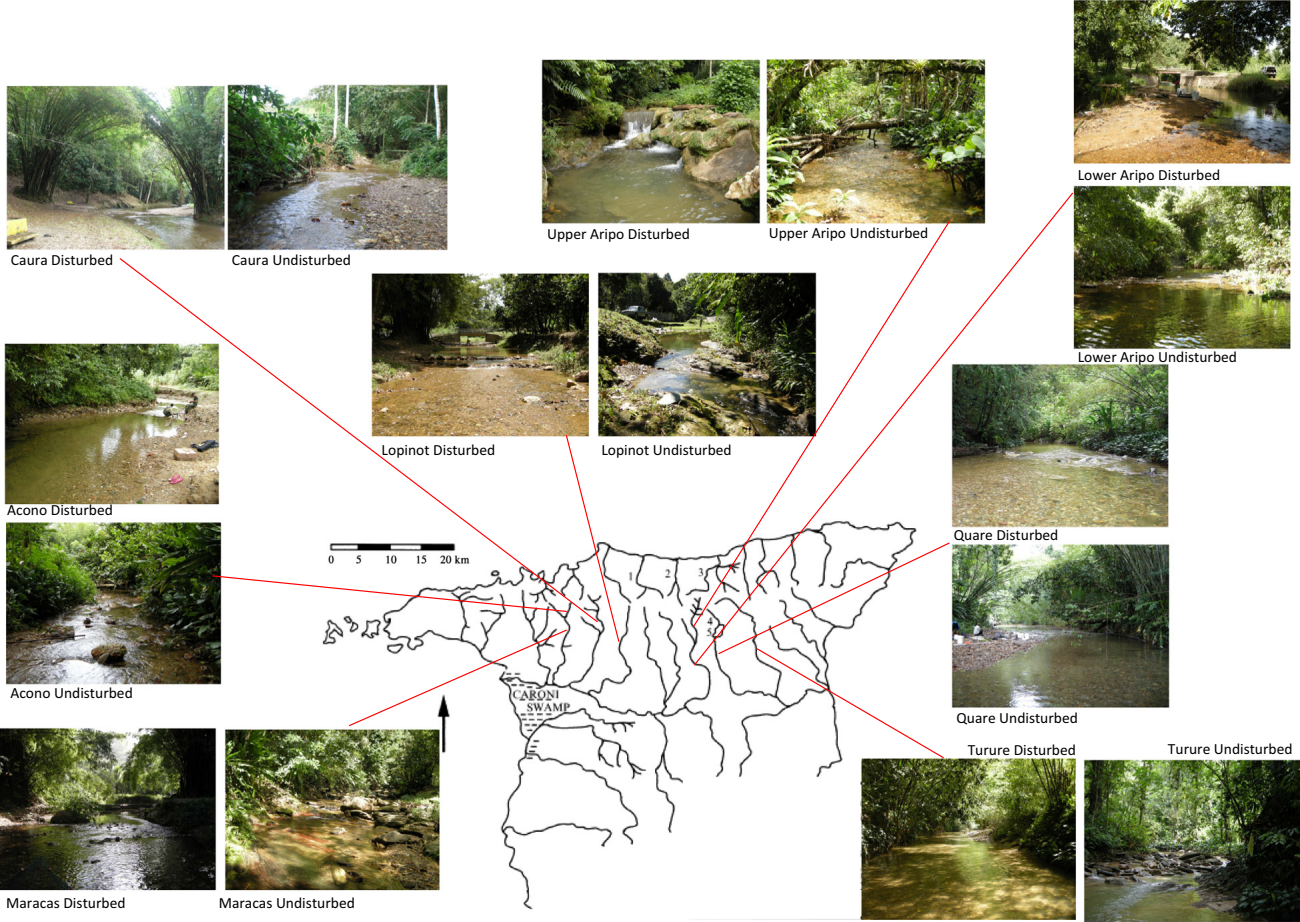
Map of Northern Range, Trinidad. Locations of pairs of sites are shown.

Eight pairs of sites in Trinidad's Northern Range were sampled (see Supp. Info. and Fig. 3). There is a strong tradition within Trinidad of choosing particular river sites for recreational activity; these sites are easily accessible by roads and have modest infrastructure in the form of shelters, barbeque sites, and refuse bins. Each pair of sites was located on the same river and was chosen to represent one recreationally used site and one nonrecreational site, the former experiencing heavy use by humans for picnicking, bathing, washing, or religious ceremonies. Otherwise the pairs of samples were as closely matched as possible in terms of stream order, flow rates, size, and isolation. Over a 28‐month period, each site was sampled 10 times, at 3 monthly intervals. Sampling was always conducted between 7:30 and 11:00. Each site consisted of a 50 m stretch of river, permanently demarcated by flagging tape.

#### Disturbance

Pairs of sites were chosen so that one in each pair had a history of heavy use for recreation. Additionally, to get a more accurate idea of the extent of recreational use, recent human use of each site was quantified by counting individual pieces of garbage on each visit and translating this onto an ordinal scale (1 = 0–5 pieces of garbage; 2 = 6–25; 3 = 26–50; 4 = 51–100; 5 = 100+). Human use of the sites varies with time of day and day of the week. As it was not possible to monitor the number of people visiting each site directly over a longer period, we decided that amount of garbage was a good proxy. From here on, this variable will be referred to as “human activity index” (HAI).

**Figure 4:**
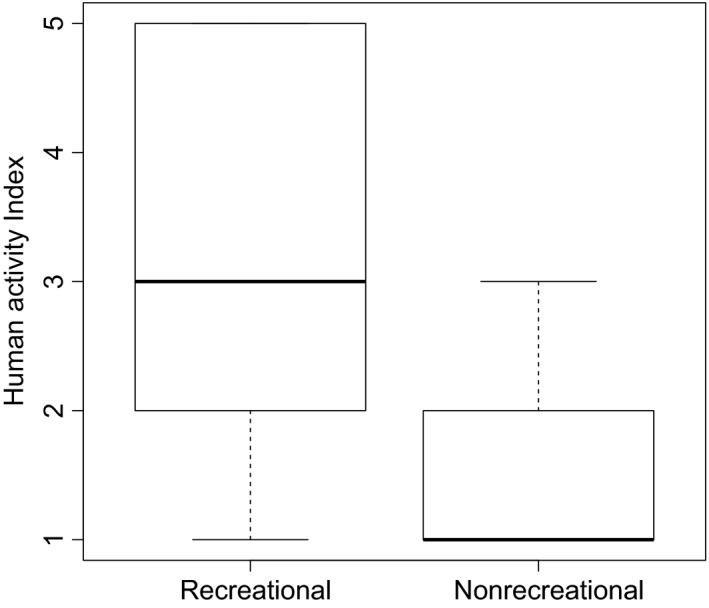
Relationship between human activity index (a measure of recent human use of a site) and whether a site is known to have been persistently used for recreation or not. Medians and interquartile ranges are shown.

A Wilcoxon rank‐sum test with continuity correction confirmed that recreational sites have significantly greater values of HAI than nonrecreational sites (*W* = 669.5; *P* < 0.001; Fig. 4). However, there exists considerable variation in extent of recent human activity among the recreational sites, suggesting that HAI gives a more precise representation of human use than the dichotomous “recreational” and “nonrecreational” division.

#### Fish

On arrival at a site, the upper and lower boundaries were blocked by seine nets to prevent any fish from moving into or out of the site during the sampling. Three methods of fishing were employed. The first involved using a two‐person seine net to fish the section. Once the whole section had been seined, electrofishing gear was used to fish the same section. Finally, a one‐person seine and dip nets were used in the shallower areas to catch juvenile fish and guppies. The three methods were always applied in this order so as not to introduce bias to the dataset. The goal was to remove all fish from the section; on the few occasions where this was not possible, a visual census was used to count the remaining individuals.

All fish caught were placed in buckets on the river bank in the shade before processing. Care was taken not to overstock the buckets and smaller species were separated from predators. Fish were identified to species and weighed (wet weight [g]) using a portable electronic balance and small tub. Guppies were not individually weighed, but size and presence of secondary sexual characteristics were used to distinguish males, females, and juveniles and totals of each were counted. Existing data from the literature were used to assign biomass to the guppy populations for females, males, and juveniles separately (Magurran 2005). After identification and weighing, all fish were returned, unharmed, to the site at which they were captured.

#### Physical site characteristics

Turbidity was measured on a scale of 1–5 (1 = clear; 5 = opaque). Oxygen levels (mg/L), pH, temperature (°C), and conductivity (*μ*S/m) were recorded at each site. At each site, three permanent transects were marked; 5, 25, and 45 m from the upstream start point.

At each transect, we recorded the width of the stream (m) and then the depth (cm) at one‐meter intervals across the breadth using a meter stick. These measurements were combined to calculate mean stream width and mean stream depth for the analysis. The flow rate (km/h) was measured using a flow meter (Global Water flowprobe or flow‐watch flow meter) in the center of each transect and one‐meter upstream and downstream of this point. The mean of these nine readings was used in the analyses. Percentage coverage of substratum types within a one‐meter belt along each transect was estimated visually. Substratum types consisted of the following: silt, sand, fine gravel, coarse gravel, cobble, small boulders, large boulders, and bedrock. For ease of estimation, only the uppermost substratum layer was included meaning that all percentages added up to 100%. Leaf litter within the same transect belt was also estimated, in addition to the 100%. In the analysis, the mean of the three transects was used for each substratum type. We estimated canopy closure using a concave spherical densiometer. The mean of nine readings per site (three along each transect) was used in the analyses.

### Statistical Methods

All statistical analyses were performed using R version 3.0.1 (RCoreTeam 2013).

#### Diversity measurements

“Hill numbers” is the collective term for a family of diversity measures, all of which measure diversity as the equivalent number of species while taking different degrees of community evenness into account (Hill 1973). They are defined by their “order” (*q*), a parameter that indicates the sensitivity of the measure to relative species abundance. Here, we calculate four levels of Hill numbers, *H*_*q*_, for our fish data: species richness (*q *=* *0), the exponential Shannon index (*q *=* *1), the reciprocal of Simpson's index (*q *=* *2), and the Berger–Parker index (*q *= ∞). From here on, they will be referred to as species richness, Shannon index, Simpson's index, and the Berger–Parker index.

#### Regression tree analysis

Using the regression tree package “Party” (Hothorn et al. 2006), we explored the importance of human recreational use relative to other environmental factors in explaining variation in each of our community properties (capacity, richness, Shannon index, Simpson's index, Berger–Parker index, and allocation). Demography (sex ratio) was not included here as the binomial structure of this data was not appropriate for analysis with the regression tree model. The explanatory variables used in the analysis were as follows: human activity index (HAI, as defined above), whether a site was classified as “recreational” or “nonrecreational” in terms of its historic human use, the river on which the site was positioned, whether the site was part of the western or eastern drainage basin, dissolved O_2_, water temperature, water conductivity, pH, turbidity, mean flow rate, mean canopy closure, mean percentage cover of silt, sand, fine gravel, coarse gravel, cobble, small boulders, large boulders, bedrock and leaf litter on the stream bottom, mean stream depth and mean stream width.

#### Generalized linear models and analysis of deviance

We used GLMs (McCullagh and Nelder 1989) to evaluate the role of anthropogenic disturbance (HAI) in accounting for variation in community capacity (biomass), community diversity (species richness and Shannon, Simpson and Berger–Parker indices), community allocation (proportion of capacity represented by guppies), and demography (sex ratio of guppies). “River” was included in the model as an additional explanatory variable so as to take account of similarities between pairs of sites.

When we assumed a non‐normal distribution for each response variable, we undertook analysis of deviance to examine the contribution of each explanatory variable (McCullagh and Nelder 1989; Venables and Ripley 2002). Although the interpretation of an analysis of deviance is similar in principle to that of an ANOVA, it is slightly more complex due to the nonorthogonality of the model. For the response variables assumed to be normally distributed, we used ANOVA. In Table 1, the values for deviance represent the variation accounted for by each factor, having eliminated the effects of those factors to the left‐hand‐side of it, but ignoring any effects of those factors to the right. A *P*‐value for each factor is also calculated; if the scale parameter of deviance is unknown, like for the gamma distribution assumed below, the *P*‐value is calculated based on the F‐statistic instead of the chi‐square statistic.

**Table 1:** Output from the analysis of deviance, with HAI (Human Activity Index) as the explanatory variable

	NULL	River	HAI	Residuals
Capacity				
Df		8	1	
Deviance		275 651	14	
Resid. Df	158	150	149	
Resid. Dev	275 737	85	72	
Pr(>F)		<2.2e‐16***	2.103e‐07***	
Richness				
Df		8	1	
Deviance		2131.43	6.79	
Resid. Df	158	150	149	
Resid. Dev	2208.10	76.67	69.88	
Pr(>Chi)		<2.2e‐16***	0.009173**	
Shannon				
Df		8	1	149
Sum Sq.		1440.25	0.50	221.68
Mean Sq.		180.03	0.50	1.49
F value		121.01	0.33	
Pr(>F)		<2e‐16***	0.57	
Simpson				
Df		8	1	149
Sum Sq.		911.85	0.58	154.12
Mean Sq.		113.98	0.58	1.03
F value		110.19	0.56	
Pr(>F)		<2e‐16***	0.460	
Berger–Parker				
Df		8	1	149
Sum Sq.		72.28	0.051	4.10
Mean Sq.		9.04	0.051	0.028
F value		328.09	1.864	
Pr(>F)		<2e‐16***	0.174	
Allocation				
Df		8	1	
Deviance		707.37	7.26	
Resid. Df	155	147	146	
Resid. Dev	929.54	222.17	2.91	
Pr(>Chi)		<2e‐16***	0.090	
Demography				
Df		8	1	
Deviance		1433.55	30.51	
Resid. Df	155	147	146	
Resid. Dev	2033	597.67	567.16	
Pr(>Chi)		<2e‐16***	3.316e‐08***	

Response variables are listed on the right. Significance levels are indicated as *P < 0.05; **P < 0.01; *** = *P* < 0.001.

##### Community capacity

Community capacity is total biomass. Taking *W* as total biomass, a GLM with a gamma distribution is fitted as

$$\text{log}\left(E\left[W\right]\right)=\sum_{j}\beta_{j}x_{j}.$$

##### Community diversity

*Species richness:* Taking *S* as richness, a GLM with Poisson distribution is fitted as

$$\text{log}\left(E\left[S\right]\right)=\sum_{j}\beta_{j}x_{j}.$$

*Simpson, Shannon, and Berger–Parker indices:* Taking *H*_*q*_ as the reciprocal of Simpson (*q *=* *2), exponential Shannon (*q *=* *1), or Berger–Parker (*q *= ∞) indices, a GLM with Gaussian distribution (a linear regression model) is fitted as

$$E\left[H_{q}\right]=\sum_{j}\beta_{j}x_{j},$$

where *H*_*q*_ is Hill's number defined as

$$H_{q}={\left(\sum_{i=1}^{s}{p_{i}}^{q}\right)}^{1/(1-q)},\;\left(q\neq 1\right)$$

For *q* = 1, Hill's number defined its limit case as $H_{1}=\lim_{q\to 1}\;H_{q}$.

##### Community allocation

Allocation is the proportion of total fish biomass represented by guppies. Taking *V* as guppy biomass, a GLM with a gamma distribution with the log link function fitted as

$$\text{log}\left(E\left[V\right]\right)=\sum_{j}\beta_{j}x_{j}+\text{log}\left(w\right),$$

where log(*w*) is log‐scaled total fish biomass and acts as an offset term whose coefficient is assumed to be one.

##### Demography

To examine the extent to which the variation of guppy sex ratio is driven by the difference between rivers, and by the disturbance condition at a site, we fitted a GLM with a binomial distribution to the guppy numerical abundance. Given the total number of guppy individuals *n*, the probability of observing *M* male individuals at a site is described by a binomial distribution:

$$\text{Pr}\left(M=m;r,n\right)=\left(\begin{array}{c} n\\ m \end{array}\right)r^{m}{\left(1-r\right)}^{n-m}$$

where *r *= *E*[*M*]/*n* is the expected sex ratio (male). The GLM models the (male) sex ratio as

$$\text{log}\left(\frac{r}{1-r}\right)=\sum_{j}\beta_{j}x_{j}$$

## Results

### Regression tree analysis

#### Community capacity

**Figure 5:**
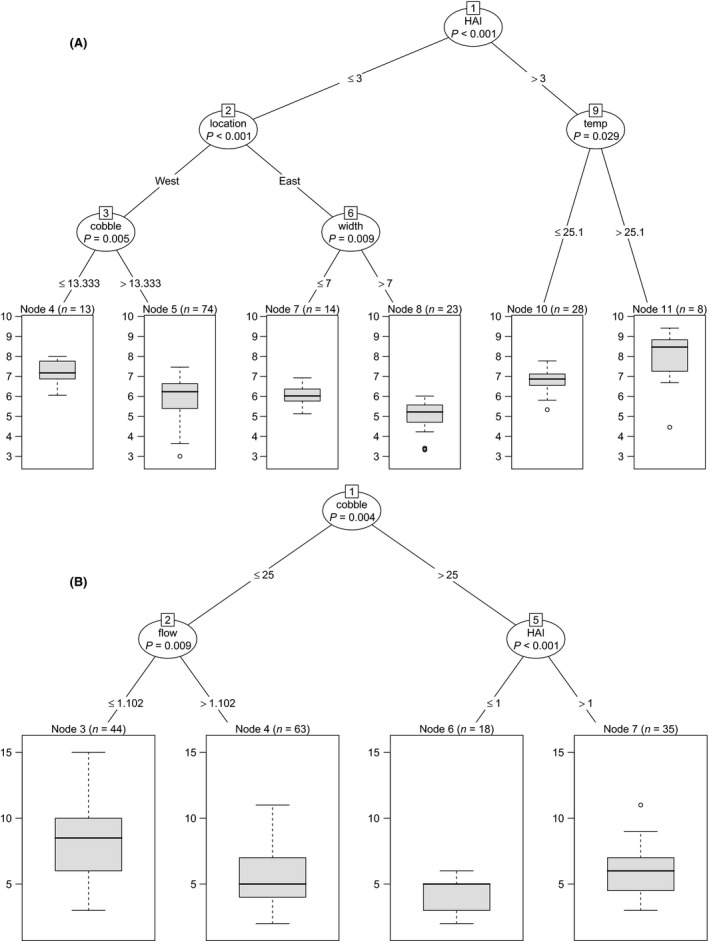
Regression trees for community (A) capacity (Ln total fish biomass) and (B) diversity (number of fish species). HAI, human activity index.

HAI stands out as the most important explanatory variable when accounting for site differences in community capacity (as measured by total biomass) (Fig. 5A). This is followed by geography (west or east drainage), substratum (% cobble), stream width, and temperature. Higher HAI is associated with greater total fish biomass. Western drainage tributaries, narrow streams, and a lower proportion of cobble substratum are also associated with greater total biomass.

#### Community diversity

*Species richness:* After cobble and flow rate, HAI is also revealed to be a significant explanatory variable when it comes to species richness, with more fish species found at sites where HAI is high. A lower percentage of cobble on the river bed was associated with greater species richness, as was a lower flow rate (Fig. 5B).

*Simpson, Shannon, and Berger–Parker indices:* HAI is not identified as an important explanatory variable in any of these regression trees, which were dominated by substratum characteristics (see appendices).

#### Allocation

When allocation was considered in the regression tree analysis, HAI did not emerge as a significant explanatory variable. Instead, just one variable emerged as important: level of dissolved oxygen in the water. Proportionally more guppies were found when dissolved oxygen levels were lower (see supplementary information).

Together, these regression trees reveal recent human activity (HAI) to be an influential factor in relation to both capacity and richness patterns across our sites, but not in relation to the Shannon, Simpson's, or Berger–Parker indices or guppy allocation patterns.

### Generalized linear models and analysis of deviance

River (i.e., which river the pair of sites was located) explained at least 70% of variation in all analyses (*P* < 0.001) (Table 1).

In terms of community capacity, the total biomass of the fish community is significantly greater at sites with higher levels of human activity (HAI) (*P* < 0.001).

Species richness is greater at sites with higher levels of HAI (*P* = 0.009).

However, variation in the Shannon, Simpson, and Berger–Parker indices between sites was not significantly linked to HAI (all *P* > 0.05).

HAI (*P* = 0.756) did not explain a significant amount of variation in proportional guppy biomass.

Proportion of male guppies is significantly lower at sites with greater levels of human activity (*P* < 0.001).

## Discussion

These results suggest a link between recreational disturbance and the ecosystem properties of a tropical stream at multiple levels: community capacity, community diversity, and demography.

### Community capacity

In our system, fish communities experiencing higher levels of anthropogenic disturbance tend to have greater total biomass. The regression tree analysis identified human impact as the most influential factor on community capacity.

A commonly observed effect of human disturbance on freshwater ecosystems is increased nutrient input (Smith et al. 1999; de Jonge et al. 2002), as a result of runoff from the surrounding land, dumping of waste and other activities associated with more heavily used sites (Lake et al. 2000). Increased nutrient input encourages raised levels of primary productivity and greater algal biomass (Smith et al. 1999). This additional biomass in primary productivity works its way up the food chain, ultimately leading to increased consumer (including fish) biomass (Hadwen and Bunn 2004; Smith and Schindler 2009). It is possible that this explains the increased total fish community capacity detected here.

An increase in community capacity has important potential implications for other ecosystem properties, including community diversity and capacity allocation, all of which are in some way constrained by, emerge from or influence capacity (Brown et al. 2001; Dornelas 2010).

### Community diversity

In addition to an increase in community capacity, we detected a shift in community diversity with increased human impact. These sites were associated with a slight, but significant, rise in species richness. This could be a consequence of increased capacity, a pattern known as the “more individuals hypothesis” (Srivastava and Lawton 1998). However, the relationship between capacity and richness is a complex one (Enquist and Niklas 2001) as it is modulated by the allocation of resources (and individuals) among species. Consequently, understanding these patterns of change requires also examining changes in species' relative abundances (see below).

Many different factors can affect the diversity patterns of freshwater systems (Matthews 1998), including habitat complexity, width/order of stream, dispersal limitation, and biogeographical factors. Human utilization trends in our rivers meant that in any pair, recreational sites were all inevitably slightly downstream of the nonrecreational site (as these tend to be the more accessible sites), but all were chosen to be as closely geographically matched as possible. Indeed, stream order varied between, but was consistent within, pairs of sites, with no sites within a pair differing by more than one “order.” Furthermore, HAI overlapped considerably between the recreational and nonrecreational sites. One of the main causes of dispersal limitation in the rivers of the Northern Range is likely to be the presence of barrier waterfalls, above which some species cannot disperse (Gilliam et al. 1993). By choosing sites that were geographically close together on each river, we minimized differences in the physical parameters of the river as well as the chance of barrier waterfalls preventing dispersal. Therefore, although we cannot completely separate any potential effects of spatial differences at our sites, HAI consistently emerges as important in both analyses.

Human activity was associated with differences in species richness patterns but not with any of the other measures of diversity that we examined from the Hill series of measures (Hill 1973). These indices all take proportional abundances of species into account to different extents, while species richness does not. This suggests species abundance distributions are not affected by this type of disturbance and adds credibility to the “more individual hypothesis” as a driver of the species richness changes we detected.

Previous studies have noted that richness can be less sensitive to disturbance than measures of evenness, which is the opposite of our finding here (Hillebrand et al. 2008). These contrasting results emphasize the need to consider multiple metrics when exploring the potential consequences of anthropogenic impacts. These results also illustrate the need to improve our understanding of the mechanisms behind these patterns of change. In particular, theoretical predictions on the effects of disturbance on species abundance distributions (and hence evenness) lag behind our predictions for changes in richness and productivity.

### Community allocation

Unlike capacity and species richness, the relative abundance of guppies does not appear to be directly linked to human recreational activity. This is in line with our finding that the diversity indices that take dominance/evenness into account did not reveal the signature of disturbance seen in capacity and richness measures. The guppy dominated these communities in terms of abundance (mean rank 1.25 ± 0.5 SD) and was well‐represented in terms of biomass (mean rank of 6.6 ± 2.5 SD; sites had a mean species richness of 10). This indicates that examining allocation of biomass to guppies is likely to be a meaningful measure of community‐level effects.

### Demography

However, when looking at the population level, we did detect a demographic shift within guppy populations at the more used sites in the analyses of deviance. Specifically, we found a small but significant difference in sex ratio, with proportionally fewer males at sites that experienced greater disturbance. It was also the case that sites with lower species richness contained proportionally fewer males. One explanation for this is that male poeciliids appear to be more sensitive to physiological stress (Snelson 1989); investment in bright coloration can reduce immunocompetence (Folstad and Karter 1992), potentially making male guppies more vulnerable to the effects of anthropogenic disturbance than females.

### Synthesis and applications

This study suggests that recreational disturbance is associated with effects at multiple levels within a tropical freshwater system: community capacity, species richness, and demography. The need for a multilevel approach when looking for effects of disturbance is further emphasized by the fact that some of the parameters examined, including three of the most commonly applied indices, were not found to be associated with differences in disturbance regime.

Capacity, richness, and allocation are known to be affected by anthropogenic disturbance (Warwick 1986; Warwick and Clarke 1994; Lake et al. 2000), but the links between these effects and population‐level effects, such as those revealed here in the guppy, are poorly studied or understood, despite being tightly connected (Enquist and Niklas 2001; Palkovacs et al. 2012). However, one possibility is that a change in guppy sex ratio may impact both higher and lower trophic levels, as female guppies are, on average, significantly larger than males and display different foraging behaviors (Dussault and Kramer 1981).

The freshwater habitats of Trinidad are rich in biodiversity and play a vital role in providing a wide range of ecosystem services, including the availability of streams for recreation (Alkins‐Koo et al. 2004; Abell et al. 2008). Users can appreciate the aesthetics of their surroundings, as well as enjoying exercise and social benefits—all of which contribute to quality of life (Bowler et al. 2010; Haines‐Young and Potschin 2010). Furthermore, use of such sites for recreation also fosters an appreciation and understanding of nature in local people, which ultimately is key to conserving habitats. Indeed, the paradox of recreational use of nature is that as well as being a source of disturbance, it is also an important ecosystem service in itself (Pinn and Rodgers 2005; Daniel et al. 2012). Our findings offer a good example of this as they show that the use of natural freshwater habitats for recreation in our system is affecting fundamental ecosystem properties (e.g., capacity), which affect patterns of diversity (e.g., richness), as well as population‐level effects (e.g., sex ratios), which in turn can have an indirect impact at the ecosystem level. However, failure to detect a signature of disturbance in allocation patterns or diversity indices in our data could be interpreted as a sign of some resilience to recreational disturbance in these communities; Hillebrand et al. (2008) suggest that changes in evenness (which should be detectable using these parameters) are more likely to have implications for ecosystem function than differences in species richness. Interpreting the consequences of the changes we have detected for the delivery of ecosystem services is a challenge for the future (Dornelas et al. 2011a).

As human populations increase, recreational use of natural habitats is set to do the same—not least for tropical streams—making it increasingly urgent that we understand the effects we have on such ecosystems. Importantly, some of the most commonly employed indices of diversity did not detect any effect of disturbance in our system, emphasizing that studies should not rely on these “classical” measures alone, especially as the implications of changes in these parameters for ecosystem function are still poorly understood. As such, we emphasize that it is essential to consider multiple levels within a community, as the effects of disturbance can be varied and interconnected. A better understanding of the ways in which ecological communities respond to disturbance is essential in light of the growing anthropogenic impacts on the natural world.

## Supporting information


**Table S1.** List of sites.

**Figure S1.** Regression tree for community allocation (proportion of total biomass represented by guppies).

**Figure S2.** Regression tree for reciprocal Simpson's index (cond=conductivity; lboulder=large boulders).

**Figure S3.** Regression tree for exponential Shannon index. (lboulder=large boulders).

**Figure S4.** Regression tree for Berger‐Parker index.
