# Supplementary material for: From species to communities: the signature of recreational use on a tropical river ecosystem
Source: Ecol Evol. 2015 Nov 12;5(23):5561–72. doi: 10.1002/ece3.1800 (PMC4813113; doi:10.1002/ece3.1800)
Supplement: Supplementary file 1 — Table S1. List of sites. [file ECE3-5-5561-s001.doc]

**SUPPLEMENTARY INFORMATION**

1. List of sites

| **River** | **Disturbance status** | **Drainage**  **(East or West)** |
| --- | --- | --- |
| Lopinot | Disturbed | W |
| Lopinot | Undisturbed | W |
| Lower Aripo | Disturbed | W |
| Lower Aripo | Undisturbed | W |
| Upper Aripo | Disturbed | W |
| Upper Aripo | Undisturbed | W |
| Acono | Disturbed | W |
| Acono | Undisturbed | W |
| Caura | Disturbed | W |
| Caura | Undisturbed | W |
| Turure | Disturbed | E |
| Turure | Undisturbed | E |
| Quare | Disturbed | E |
| Quare | Undisturbed | E |
| Maracas | Disturbed | W |
| Maracas | Undisturbed | W |

1. **Additional Regression trees for:**
2. Allocation
3. Simpson’s
4. Shannon
5. Berger-Parker
6. Demography
