# Supplementary figures and images for: From species to communities: the signature of recreational use on a tropical river ecosystem
Source: Ecol Evol. 2015 Nov 12;5(23):5561–72. doi: 10.1002/ece3.1800 (PMC4813113; doi:10.1002/ece3.1800)

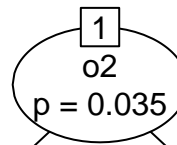

$\leq 6.82$

$> 6.82$

Node 2 (n = 29)

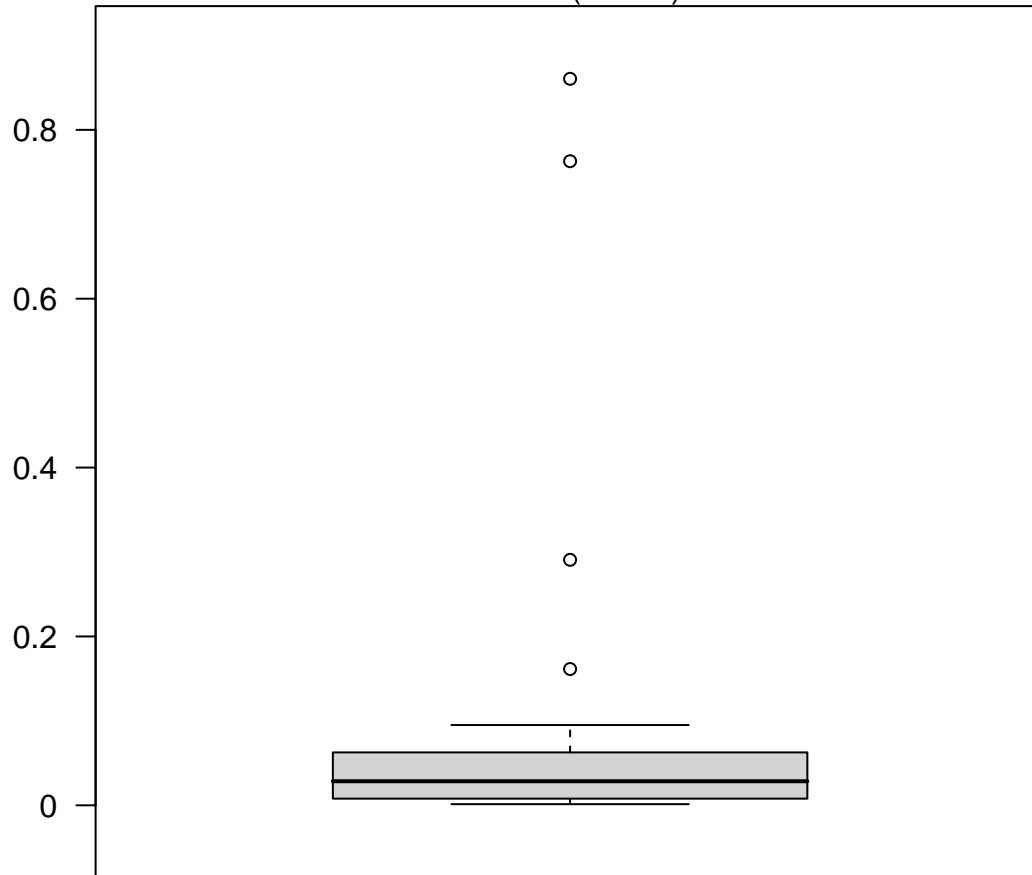

Node 3 (n = 131)

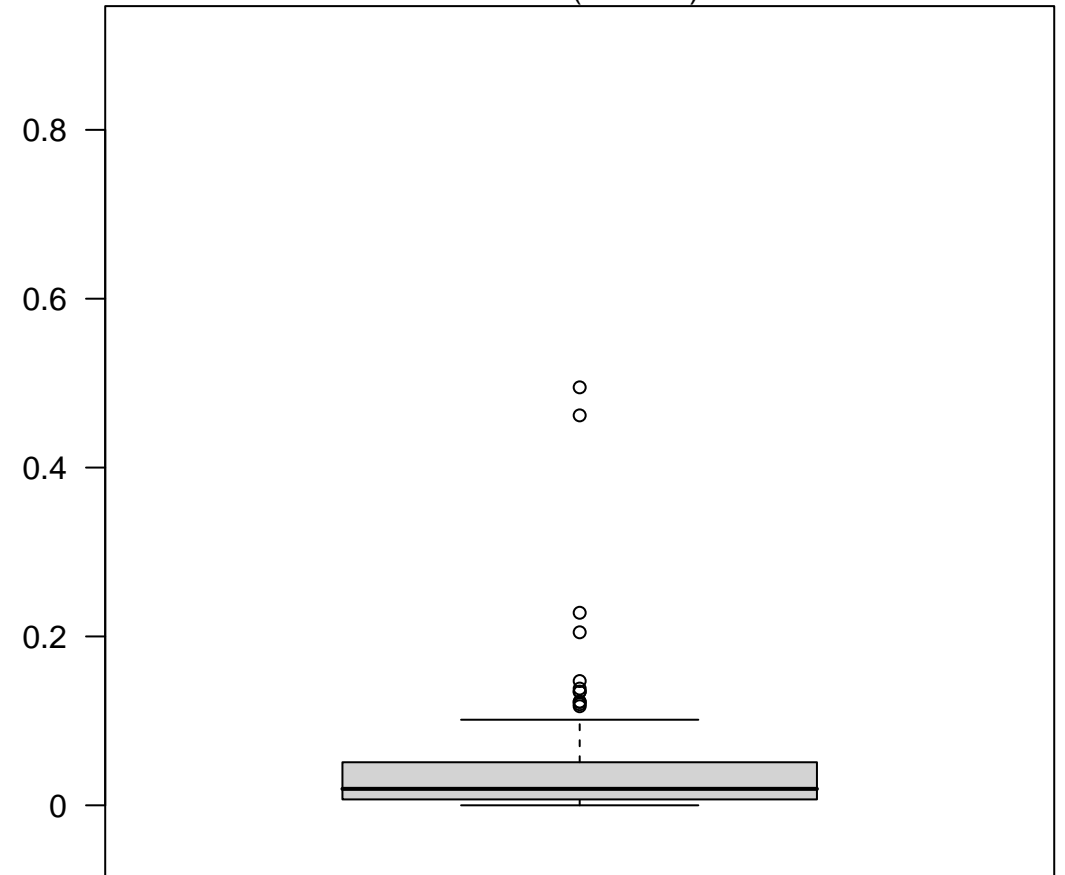

Supplement: Supplementary file 2 — Figure S1. Regression tree for community allocation (proportion of total biomass represented by guppies). [file ECE3-5-5561-s002.pdf]

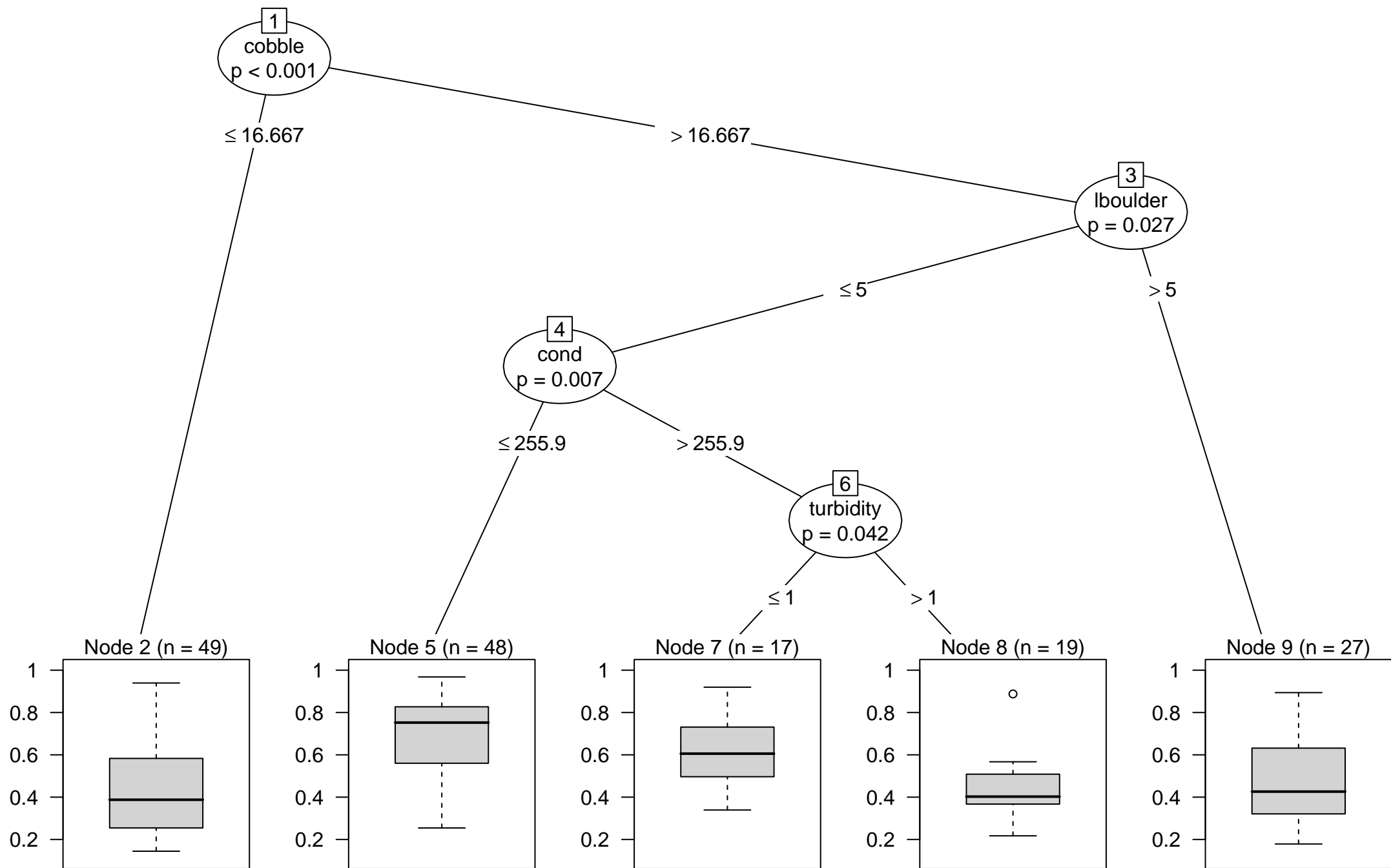

Supplement: Supplementary file 3 — Figure S2. Regression tree for reciprocal Simpson's index (cond=conductivity; lboulder=large boulders). [file ECE3-5-5561-s003.pdf]

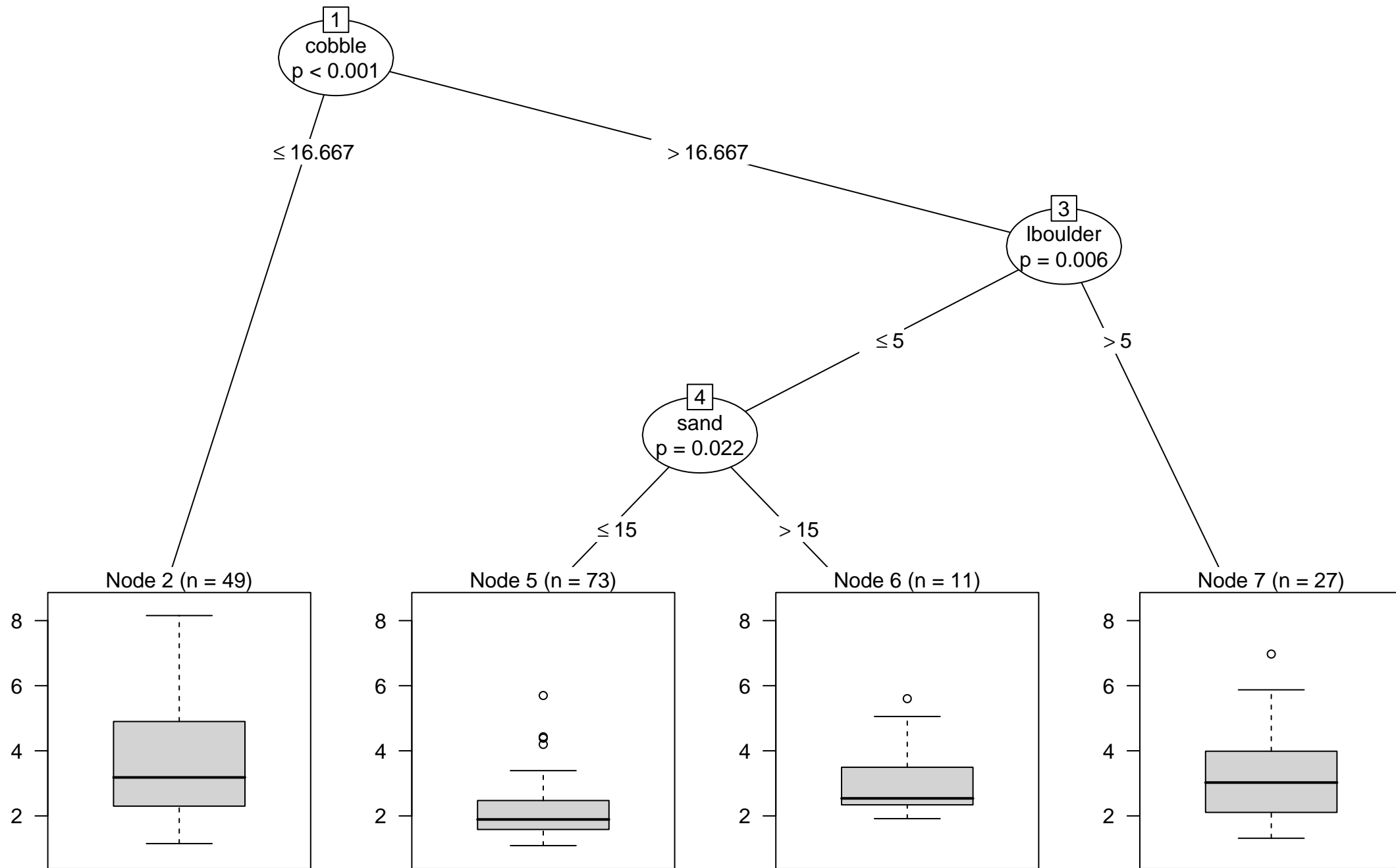

Supplement: Supplementary file 4 — Figure S3. Regression tree for exponential Shannon index. (lboulder=large boulders). [file ECE3-5-5561-s004.pdf]

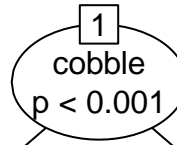

$\leq 13.333$

$> 13.333$

Node 2 (n = 41)

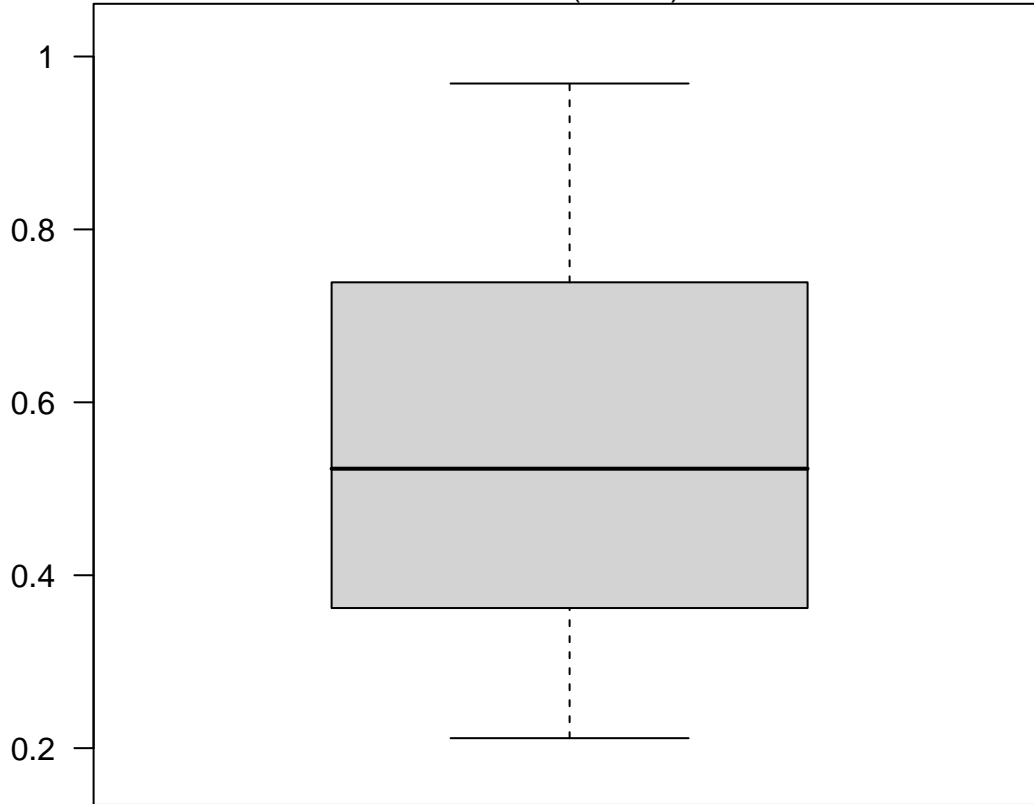

Node 3 (n = 119)

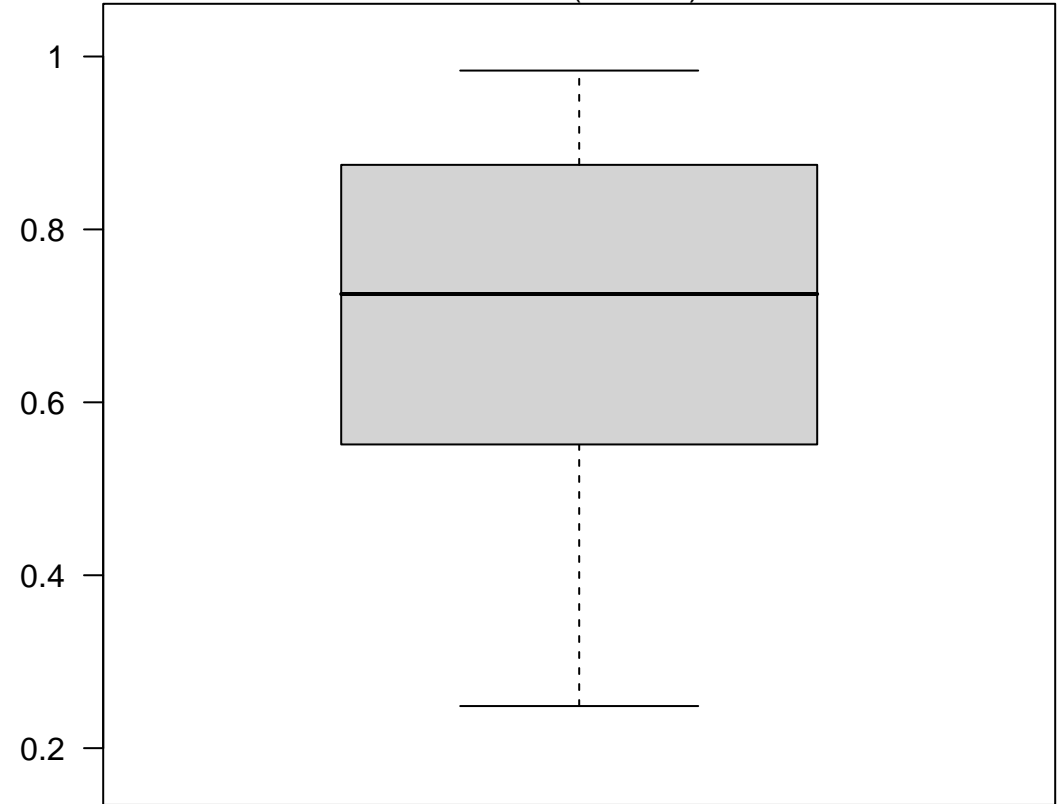

Supplement: Supplementary file 5 — Figure S4. Regression tree for Berger‐Parker index. [file ECE3-5-5561-s005.pdf]
